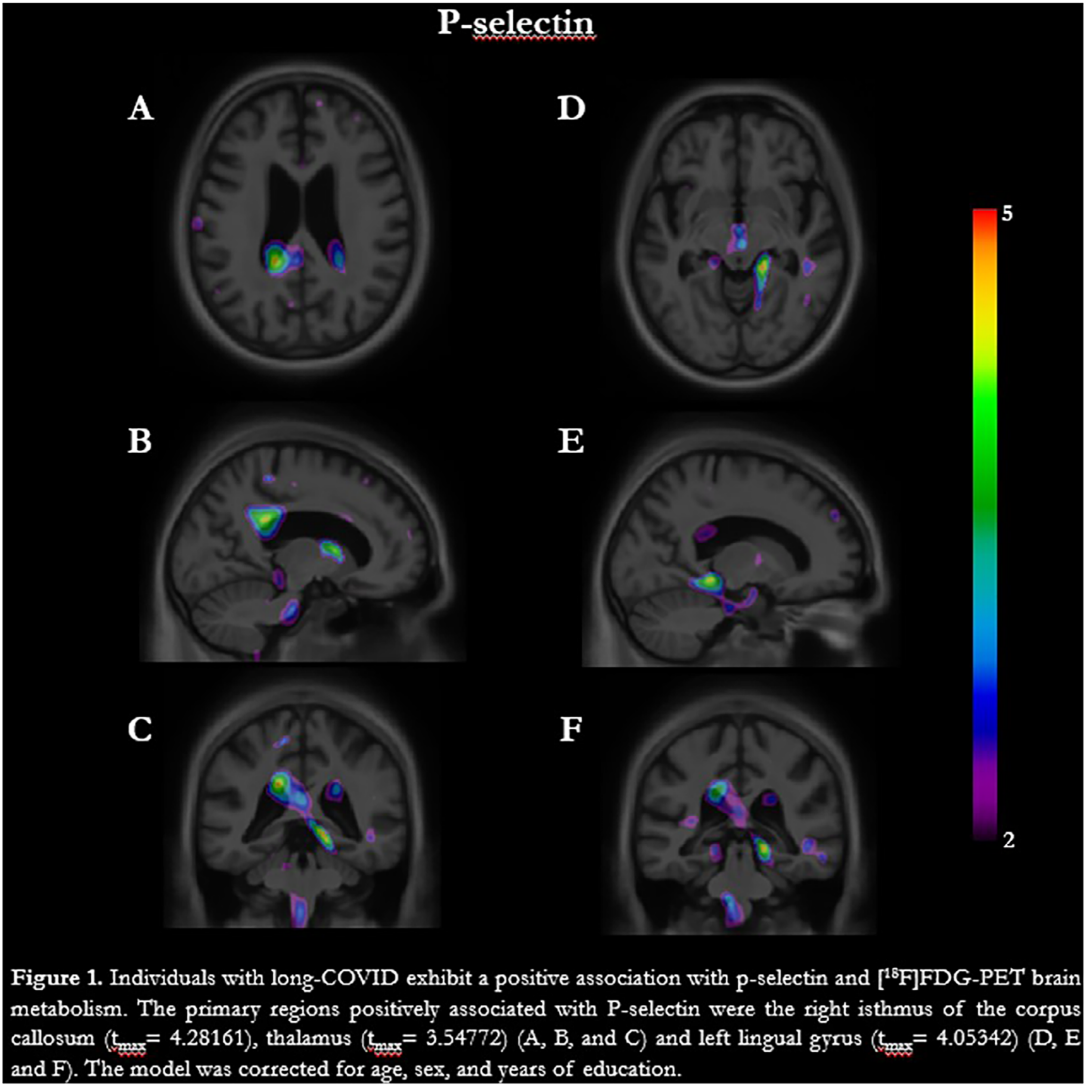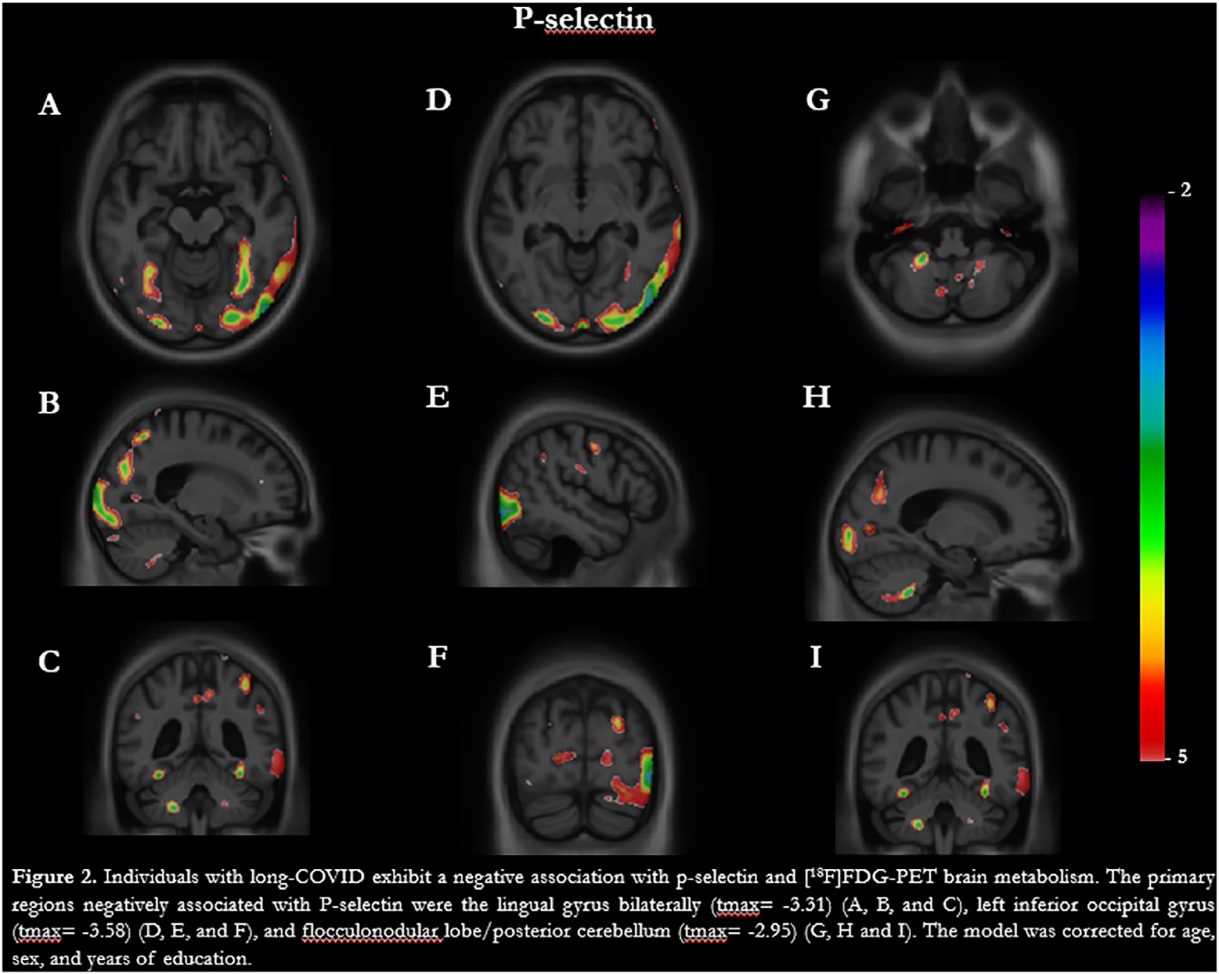# Association between blood biomarkers of vascular endothelial dysfunction and brain glucose metabolism in long COVID

**DOI:** 10.1002/alz.093430

**Published:** 2025-01-09

**Authors:** Ana Paula Bornes da Silva, Luiza Santos Machado, Marina Siebert, Maiele Dornelles Silveira, Wyllians Vendramini Borelli, Joana Emilia Senger, João Pedro Uglione da Ros, Arthur Viana Jotz, Guilherme Bastos de Mello, Matheus Fakhri Kadan, Graciane Radaelli, João Pedro Ferrari‐Souza, Marco Antônio de Bastiani, Guilherme Povala, Cristina Sebastião Matushita, Ricardo Benardi Soder, Artur Francisco Schumacher‐Schuh, Tharick A. Pascoal, Diogo O. Souza, Mychael V Lourenco, Daniele de Paula de Paula Faria, Artur Martins Coutinho, Jaderson Costa daCosta, Débora Guerini de Souza, Eduardo R. Zimmer

**Affiliations:** ^1^ Federal University of Rio Grande do Sul, Brazil, Porto Alegre, RS Brazil; ^2^ Federal University of Rio Grande do Sul, Porto Alegre, Rio Grande do Sul Brazil; ^3^ Clinical Hospital of Porto Alegre, Porto Alegre, Rio Grande do Sul Brazil; ^4^ Federal University of Rio Grande do Sul, Brazil, Porto Alegre, Rio Grande do Sul Brazil; ^5^ Lutheran University of Brazil, Canoas, Rio Grande do Sul Brazil; ^6^ Pontifical Catholic University of Rio Grande do Sul, Porto Alegre, Rio Grande do Sul Brazil; ^7^ Pontifícia Universidade Católica do Rio Grande do Sul, Porto Alegre, Rio Grande do Sul Brazil; ^8^ Brain Institute, RS, Porto Alegre, Rio Grande do Sul Brazil; ^9^ University of Pittsburgh, Pittsburgh, PA USA; ^10^ Brain Intitute, RS, Porto Alegre, Rio Grande do Sul Brazil; ^11^ Federal University of Rio Grande do Sul (UFRGS), Porto Alegre, RS Brazil; ^12^ Federal University of Rio de Janeiro, Rio de Janeiro, Rio de Janeiro Brazil; ^13^ University of São Paulo Medical School, São Paulo, São Paulo Brazil

## Abstract

**Background:**

COVID‐19, identified as the greatest health concern of the century, is associated with vascular inflammation and endothelial activation, resulting in multisystemic damage, including to the central nervous system (CNS). Recent investigations indicate a link between endothelial dysfunction, neurological changes, and the development of the so‐called long‐COVID. Molecules expressed in the endothelium such as P‐selectin, E‐selectin, and VEGF‐A, increased under inflammatory injury, may be associated with conditions like brain injuries and neurodegenerative diseases. These markers may remain altered in the organism for months after the acute episode of COVID and may be related to long‐term neurological disorders. This study aims to identify associations between biomarkers of endothelial dysfunction and brain glucose metabolism in individuals with long‐COVID.

**Methods:**

A total of 39 individuals presenting with long‐COVID and 10 healthy controls (HC) underwent brain [^18^F]FDG‐PET imaging. Standardized uptake value ratio maps were generated using the [^18^F]FDG‐PET global mean. Using an ELISA Multiplex assay, we measured P‐selectin, E‐selectin, and VEGF‐A in plasma samples. Voxel‐wise linear regressions models accounting for age, gender, and years of education were used to evaluate the association between the interaction biomarker*group and the brain glucose metabolism.

**Results:**

The analyses showed that the interaction between P‐selectin and group presents positive association clusters in the right isthmus of the corpus callosum, thalamus, and left lingual gyrus (Figure 1). In this model, we also observed negative association clusters in the lingual gyrus bilaterally, the left inferior occipital gyrus, and the flocculonodular lobe (posterior cerebellum) (Figure 2). The linear regression analysis with the interaction between VEGFA or E‐selectin and group did not present a significant association with [^18^F]FDG‐PET signal.

**Conclusion:**

The results provide preliminary insights into the complex interactions between endothelial dysfunction and cerebral glucose metabolism in long‐COVID. The regions presenting significant associations are involved in cognition, memory, sensory and motor function, language and visual processing, indicating a potential widespread brain vascular response. Additional studies are needed for a comprehensive understanding of these relationships in long‐COVID.